# Predicting the plastic biodegradation potential within microbial lineages and across global ecosystems

**DOI:** 10.1099/mgen.0.001814

**Published:** 2026-08-11

**Authors:** Harmony Douwes, Zuzanna Dutkiewicz, Christian Rinke

**Affiliations:** 1 Australian Centre for Ecogenomics, School of Chemistry and Molecular Biosciences, The University of Queensland, St. Lucia, QLD, Australia; 2 Department of Microbiology, The University of Innsbruck, 6020 Innsbruck, Austria

**Keywords:** archaea, environment, enzymes, Hidden Markov Models, metagenomics, micro-organisms, plastic biodegradation, pollution

## Abstract

Plastic waste pollution is a global issue that threatens biodiversity and human health. Current plastic waste management practices are not sufficient to keep up with increasing plastic production rates. Microorganisms have the capacity to degrade different types of bio-based and synthetic plastics through enzymatic reactions, offering an alternative solution to traditional plastic recycling techniques. A limited number of plastic-degrading enzymes have been identified, sequenced and characterized; however, studies exploring the distribution of homologues of these enzymes across habitats and microbial taxa have remained scarce. Here, we applied analytical techniques to search for genes encoding potential plastic-degrading enzymes in environmental metagenome datasets and genomes of the Genome Taxonomy Database (GTDB) to explore the geographic and taxonomic distribution patterns of plastic-degrading microorganisms. Hidden Markov Models (HMMs) were constructed from amino acid sequences of known, experimentally verified and putative plastic-degrading enzymes. The HMMs were applied to landfill, soil, river, lake and ocean metagenomes and all archaeal and bacterial genomes in the GTDB. An abundance of hits was discovered across aquatic and terrestrial metagenomes with the majority occurring in polluted rivers, polar oceans and deep ocean samples. GTDB hits were mainly consistent with known plastic-degrading microbial lineages, while also revealing potential plastic-degrading archaeal taxa. The results of this study may be able to assist in the discovery of novel plastic-degrading enzymes for application in plastic waste biodegradation solutions.

**Impact Statement**

Plastic pollution is a growing global threat, yet the diversity and distribution of microorganisms capable of degrading plastics remain poorly understood. This study provides a broad, data-driven assessment of genes encoding potential plastic-degrading enzymes across major ecosystems and microbial lineages by applying curated Hidden Markov Models to extensive metagenomic and genomic datasets. We reveal that putative plastic-degrading capacity is far more widespread across GTDB taxa than previously recognized, spanning diverse bacterial groups and, unexpectedly, archaeal taxa. High hit densities in polluted rivers, deep-sea environments and polar oceans highlight habitats where natural plastic turnover may be most active. By identifying both known degraders and previously uncharacterized genera with strong biodegradation potential, this work offers a targeted foundation for experimental validation and enzyme discovery. The study advances the field by providing a scalable framework for detecting genes encoding plastic-degrading enzymes and guiding future laboratory efforts towards the most promising taxa and environments. These insights support the development of biologically based strategies for mitigating plastic pollution and represent a meaningful step towards harnessing microbial diversity for sustainable plastic waste management.

## Data Summary

The data generated or analyzed during this study are included in this published article and its supplementary information files, except for the HMMs, seed enzyme fasta sequences and additional seed enzyme and HMM information, which are all provided at https://doi.org/10.5281/zenodo.15480170. BioProject and accession numbers for the metagenomic datasets are listed in Table S3.

## Introduction

For a material invented in the mid-1900s, it’s incredible how plastic has become so irrevocably integrated into modern-day industries from construction to human health [1]. Its lightweight and versatile properties combined with the low cost to manufacture have contributed to large-scale production and accumulation of plastic waste. According to the OECD, over 460 million tonnes of primary plastic were manufactured in 2021 alone, with approximately one third used to produce single-use packaging [2]. Global plastic recycling rates continue to lag at only 15–18% of all plastic waste due to cost and compliance challenges [1, 2], while one quarter to one third of plastic waste is inadequately managed in uncontrolled dumps, landfills or littered [2, 3]. Mismanaged waste becomes plastic pollution and is carried through air currents and water transport pathways [3]. Since a large proportion of the world’s population lives in coastal regions or along river tributaries, the ultimate destination for most plastic pollution is the ocean, where it can harm marine wildlife through entanglement or ingestion [4, 5]. The partial disintegration of ocean plastics, primarily due to UV exposure [6], results in microplastic (<5 mm diameter) and nanoplastic (<0.1 µm) particles that may pose increased risks since they can infiltrate and accumulate at the base of the food chain. For instance, a meta-analysis of 50 publications found that filter-feeding molluscs contained the highest amounts of microplastic contamination relative to fish and crustaceans [7]. Furthermore, microplastic particles have been discovered dispersed in extreme and remote environments such as the deep ocean [8] and Antarctic snow [9]. Recently, plastic particles have even been detected in human blood [10], testis [11] and brain tissue [12]. Since modelling predicts that plastic production is likely to increase over the next several decades [13], there is an urgent need for alternative and effective solutions to sustainable plastic production and waste management.

Plastic materials are composed of repeating monomer units that form high-molecular-weight polymer chains containing a carbon-based backbone. Differences in the chemical structures of these backbones result in a wide range of plastic types. The specific monomer typically influences the three-dimensional polymer structure, which in turn can impact the properties of the plastic. Chemical additives, including UV stabilizers or plasticizers, such as bisphenol A and dibutyl phthalate (DBP) [14], can be used to further alter plastic material attributes. Nowadays, the most common plastic types include polyethylene (PE), polypropylene (PP), polyethylene terephthalate (PET), polyvinyl chloride (PVC) and polystyrene (PS). Together, these five plastics make up more than 80% of the global plastic production [1]. All are typically manufactured from synthetic raw materials, i.e. crude oil, natural gas or coal. While bio-based plastics derived from renewable resources are emerging, their production has remained relatively low, contributing less than 2% to the global plastic market [1]. Examples include polylactic acid (PLA), polyhydroxyalkanoates (PHAs) and bio-based analogues of standard synthetic plastics (e.g. Bio-PET). Although derived from renewable resources, not all bio-based plastics are readily biodegradable. For example, Bio-PE, Bio-PA and Bio-PET are not considered to be biodegradable [15], while PLA can only compost under very specific conditions which may not occur in nature [16].

Plastic materials can be broken down through both abiotic and biotic degradation processes. Abiotic processes include physical fragmentation, thermal, UV and chemical breakdown, while biotic degradation primarily involves depolymerization through the activity of microbial enzymes [6, 17]. Some plastic types are more readily digested through enzymatic activity than others. For example, aliphatic and aromatic polyesters, including PET and PHA, are susceptible to enzymatic hydrolysis, whereas polymers with non-hydrolysable carbon backbones, such as PS and PE, are generally much more resistant to cleavage [17]. Abiotic degradation can accelerate biotic degradation (and vice versa) through both chemical modifications, i.e. exposing reactive groups, and by increasing the surface area of the plastic particles through cracking and fragmentation [17].

Microorganisms capable of degrading different plastic types have been identified in a range of ecosystems from hot springs [18] to the digestive tracts of insects [19, 20]. One of the best characterized plastic biodegradation pathways is the PETase and MHETase enzyme system of a bacterium isolated from a PET bottle recycling facility, *Piscinibacter sakaiensis* (syn. *Ideonella sakaiensis*) [21]. The *P. sakaiensis* population could break down PET film and convert it into carbon dioxide through an efficient two-stage process involving the formation of mono(2-hydroxyethyl) terephthalic acid followed by terephthalic acid monomers [21, 22]. Besides *P. sakaiensis*, a range of microorganisms have been demonstrated to enzymatically degrade plastic materials, including the genera *Pseudomonas* [23–27], *Thermobifida* [28–31], *Comamonas* [32, 33] and *Aspergillus* [34–36]. Some bacterial species are capable of both degradation and synthesis of aliphatic polymer molecules like PHA, including polyhydroxybutyrate (PHB), utilizing them as a carbon storage mechanism [37]. A review of over 1,450 publications found that plastic-degrading microorganisms have been reported within 5 out of 31 bacterial and 3 out of 11 fungal phyla within the NCBI database [38]. However, no plastic-degrading archaeal taxa have yet been identified [38]. Despite the extensive research documenting microbial plastic degradation, the identification and sequencing of plastic-degrading enzymes have remained relatively limited.

A study by Zrimec *et al*. [39] explored global distribution patterns of potential plastic-degrading genes by constructing a set of Hidden Markov Models (HMMs) to screen metagenomic datasets. Zrimec *et al*. [39] found genes encoding plastic-degrading enzymes to be distributed across both soil and ocean ecosystems using the Tara Oceans and Global Soil Microbiome datasets. Danso *et al*. [40] also developed and utilized HMMs to identify and experimentally verify a subset of putative PET-degrading enzymes within the Integrated Microbial Genome database. The genes of 4 of the 349 identified amino acid sequences were heterologously expressed, and all demonstrated PET- or PCL-degrading ability on an agar plate model substrate [40]. More recently, Kumar *et al*. [41] developed a single PET-degrading enzyme HMM from amino acid sequences described in the plastics-active enzymes database (PAZy) and applied this to the MGnify metagenomic database. HMM-recovered predicted amino acid sequences were analyzed using motif identification, 3D modelling and molecular docking techniques to shortlist three putative PET-degrading enzyme candidates with predicted PET-binding affinities comparable to the well-characterized IsPETase [41]. HMMs have also been applied to other metagenomic mining projects, such as for the identification of recalcitrant biopolymer degradation enzymes [42], viruses [43] and antimicrobial resistance genes [44]. Although acknowledged to be an effective way to identify novel plastic-degrading enzymes [45, 46], there are few other examples which have applied HMMs in this exact manner.

In this study, we used an HMM-based pipeline to screen for genes encoding a curated set of plastic-degrading enzymes across a broad collection of aquatic and terrestrial metagenomes, as well as the Genome Taxonomy Database R214 (GTDB) [47]. For the remainder of the manuscript, we will refer to these HMMs as ‘plastic-degrading enzyme HMMs’. Twenty-two different target substrates were included, encompassing bio-based, synthetic, biodegradable and recalcitrant polymers and plastic additives. This is the first study to explore the plastic-degrading potential of all genomes within GTDB, in combination with a screen of environmental metagenomes. Our goal was to guide efforts in identifying novel plastic-degrading taxa and their potential environmental niches, with the ultimate aim of discovering enzyme sequences for applications in plastic waste biodegradation systems.

## Methods

### Construction of HMMs

HMMs were constructed from a curated set of amino acid sequences derived from enzymes with published evidence of plastic-degrading activity. The PAZy database [48] was used to identify enzyme leads, but only the primary literature was used to establish evidence of plastic degradation rather than presence in the PAZy database alone. Both experimentally verified and putative plastic-degrading enzymes were considered. Acceptable direct experimental evidence of plastic degradation included model substrate depletion with the isolated or recombinantly expressed enzyme or demonstration of loss of function after targeted gene knock-out. For enzymes without direct evidence of plastic degradation ability, the inclusion criteria were (i) direct evidence of plastic degradation at the organism level and either (ii) evidence of gene upregulation during plastic degradation or (iii) sequence homology to known and experimentally verified plastic-degrading enzyme(s). A total of 145 different amino acid sequences were identified and compiled. This list included sequences for 141 experimentally verified [18, 21, 23–36, 40, 49–113] and 4 putative [20, 114, 115] plastic-degrading enzymes encompassing 22 different plastics and additives (Tables 1 and S1, available in the online Supplementary Material). While including putative plastic-degrading enzymes entails a risk of false positives, the inferred evidence of their plastic-degrading activity was deemed strong enough to warrant retention (Table S1). Notably, two of the inferred plastic-degrading enzymes were the only representatives in the dataset linked to degradation of recalcitrant PS. The other two were a putative PE- and a putative DBP-degrading enzyme.

**Table 1.** Plastic targets included in this study. Each plastic was characterized by the molecular category and based on its source. Note that some plastics can be created from synthetic or bio-based sources

Plastic	Abbreviation	Molecular category	Plastic source
Dibutyl phthalate	DBP	Ester	Synthetic
Polyamide (=nylon)	PA	Amide	Synthetic
Polybutylene adipate terephthalate	PBAT	Ester	Synthetic
Polybutylene succinate	PBS	Ester	Synthetic/bio-based
Polybutylene succinate-co-butylene adipate	PBSA	Ester	Synthetic/bio-based
Polycaprolactone	PCL	Ester	Synthetic
Polyethylene	PE	Carbon	Synthetic
Polyethylene glycol	PEG	Ether	Synthetic
Polyethersulfone	PES	Ether and sulphonyl	Synthetic
Polyethylene terephthalate	PET	Ester	Synthetic
Polyhydroxyalkanoate	PHA	Ester	Bio-based
Polyhydroxybutyrate	PHB	Ester	Bio-based
Polyhydroxyoctanoate	PHO	Ester	Bio-based
Polyhydroxyvalerate	PHV	Ester	Bio-based
Poly(3-hydroxybutyrate-co-3-hydroxyvalerate)	PHBV	Ester	Bio-based
Phthalate	-	Ester	Synthetic
Polylactic acid	PLA	Ester	Bio-based
Cross-linked elastomer	CE	Ester	Bio-based
Polystyrene	PS	Carbon	Synthetic
Polyurethane	PU	Urethane	Synthetic/bio-based
Polyvinyl alcohol	PVA	Carbon	Synthetic
Terephthalate	TP	Ester	Synthetic

**Fig. 1.**
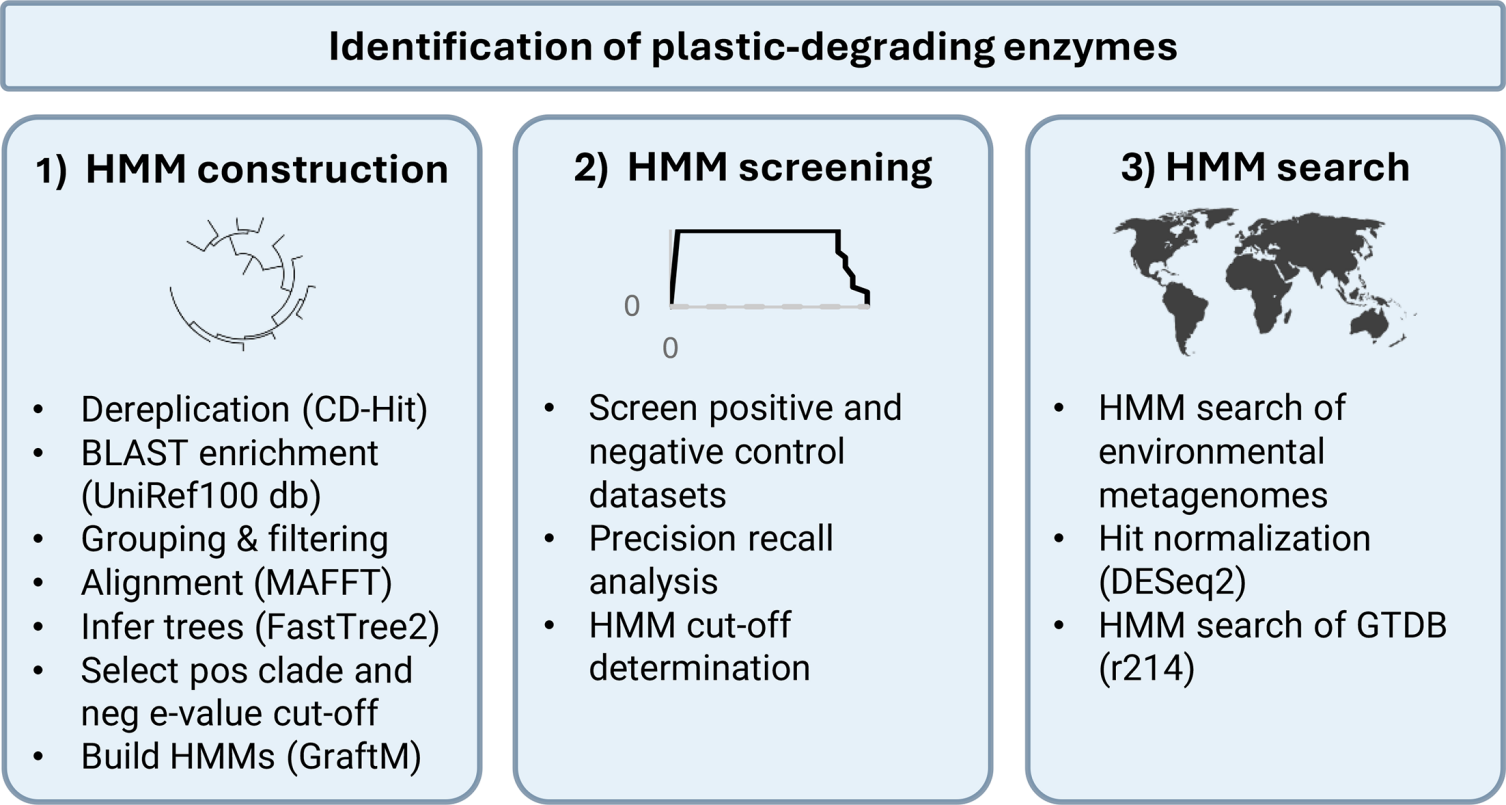
Methods for generation and application of HMMs constructed from the experimentally verified or putative plastic-degrading seed enzyme amino acid sequences. Note that the initial set of 145 enzymes was dereplicated, based on their amino acid sequences, to 112 seed enzymes. This number was further reduced to 65 through phylogenetic inference, from which 53 HMMs were reconstructed.

After compilation, the set of plastic-degrading enzyme amino acid sequences was dereplicated by clustering at 95% identity using CD-Hit [116] version 4.8.1. This reduced the total number of sequences from 145 to 112. Next, the dataset was enriched by a blast (version 2.9.0+) search of the seed enzyme sequences against the UniRef100 database (downloaded 17^th^ September 2022) [117] using an e-value cut-off of 1e-10 and an 80% minimum query coverage threshold. Manual curation of the blast results was performed to identify overlapping datasets and group them accordingly. For instance, if a seed enzyme sequence was found to be included in the blast search results for another seed enzyme sequence, the blast results for both were combined into a ‘grouped’ dataset and dereplicated. Next, datasets containing more than 5,000 sequences were clustered at 70% identity using CD-Hit [116] before aligning. If there were still >5,000 sequences remaining after clustering, blast hits with ≤40% identity to the seed sequence(s) were filtered out. The sequence datasets were then aligned using MAFFT [118] version 7.505 and either default parameters or 100 maximum iterations. Phylogenetic trees were inferred from the aligned sequence datasets using FastTree2 [119] version 2.1.9 with default parameters. Grouped datasets were reassessed at this stage. If the original seed enzyme sequences were within the same branch of the phylogenetic tree, the group was maintained. However, if the seed enzyme sequences were located on distinct branches, the group was rejected, and separate phylogenetic trees were inferred from the original, ungrouped sequences. This approach yielded a condensed set of 65 non-redundant phylogenetic trees. The full workflow for aligning the sequences and inferring phylogenetic trees is detailed in Fig. 1. See Fig. S1 for illustrative examples.

Once the phylogenetic trees were constructed, sequences within the same monophyletic clade as the seed enzyme sequence(s) were chosen as input for the generation of plastic-degrading enzyme HMMs. The HMM input sequences were manually selected based on the tree topology and informed by the blast e-value and percent identity results for each sequence within the tree. Negative control sequences were also identified using the phylogenetic trees; a maximum e-value threshold was established for each tree, such that the sequences with a blast e-value above this cut-off were sufficiently distinct from the monophyletic clade containing the seed enzyme sequence(s). The negative control sequences were used to assess precision and recall of the generated HMMs as described in *HMM cut-off selection*. The selected HMM input sequences were dereplicated by clustering again at 95% identity before generating HMMs using GraftM [120] version 0.14.0 with default parameters.

### HMM cut-off selection

Recall and precision of the plastic-degrading enzyme HMMs were assessed using positive and negative control datasets specific to each HMM. The negative control datasets were composed of homologous amino acid sequences which had a blast e-value score above the negative control threshold established for each phylogenetic tree as described above in *Construction of HMMs*. The positive control datasets included all amino acid sequences within the monophyletic clade containing the seed enzyme sequence(s), i.e. all HMM input sequences plus the sequences removed via clustering. This approach was deemed acceptable, since the optimal HMM cut-offs calculated using all positive sequences were strongly correlated (R^2^=0.95) (Fig. S2) with the cut-offs calculated using the positive sequences removed during the clustering step (and therefore not used to generate the HMMs directly).

Precision versus recall curves were generated for each plastic-degrading enzyme HMM by ranging the HMM search e-value cut-off from 1 to 1e-300. Precision and recall values were used to calculate the maximum F1 measure, thereby determining the optimal e-value cut-off specific to each HMM (Table S2). Only models with greater than 95% precision were accepted into the final set of 53 plastic-degrading enzyme HMMs.

### Metagenomic datasets

Ocean, river, lake, soil and landfill metagenomic datasets were searched for genes encoding potential plastic-degrading enzymes. The Tara Oceans Project [121], GEOTRACES [122], Malaspina Deep Ocean Project [123], Polar Oceans [124] and 2014 Ocean Sampling Day [125] metagenomic datasets were included for a comprehensive snapshot of different geographic regions and oceanic ecosystems. For the river metagenomes, the Amazon River [126], Brisbane River [127], Ganges River [128], Yangtze River [129] and Canadian river systems [130] were selected to represent a breadth of different habitats with varying levels of anthropogenic influence. Freshwater lakes [131], global soil microbiome [132] and landfill leachate samples [133] were also included. BioProject and accession numbers for the metagenomic datasets are listed in Table S3. If the samples were size-fractionated, only those corresponding to ≥0.2 µm were included in the analysis.

For the Tara Oceans Project, Brisbane River and GEOTRACES datasets, the available published assemblies were used. For all other metagenomic datasets, reads were quality-checked with FastQC [134] version 0.11.9 and assembled with MEGAHIT [135] version 1.2.9 using default parameters.

### HMM searching and hit score normalization

Using GraftM [120], the final 53 plastic-degrading enzyme HMMs and their specific e-value cutoffs (Table S2) were applied to search the GTDB [47] R214 reference genomes for potential plastic-degrading bacterial and archaeal species. Environmental metagenomes from ocean, river, lake, soil and landfill samples were also screened. Taxonomy was assigned to each hit by GraftM during HMM searching, based on the NCBI taxonomy associated with the HMM input sequences. All environmental hit counts were then normalized with DESeq2 v1.40 [136] (applying mean fit type and variance stabilizing transformation) to yield relative hit scores as a measure of abundance of plastic-degrading gene hits. After normalization, mean hit scores were calculated across samples to compare environmental features of interest, such as ocean vertical depth zone.

## Results

### Plastic-degrading enzymes

**Fig. 2.**
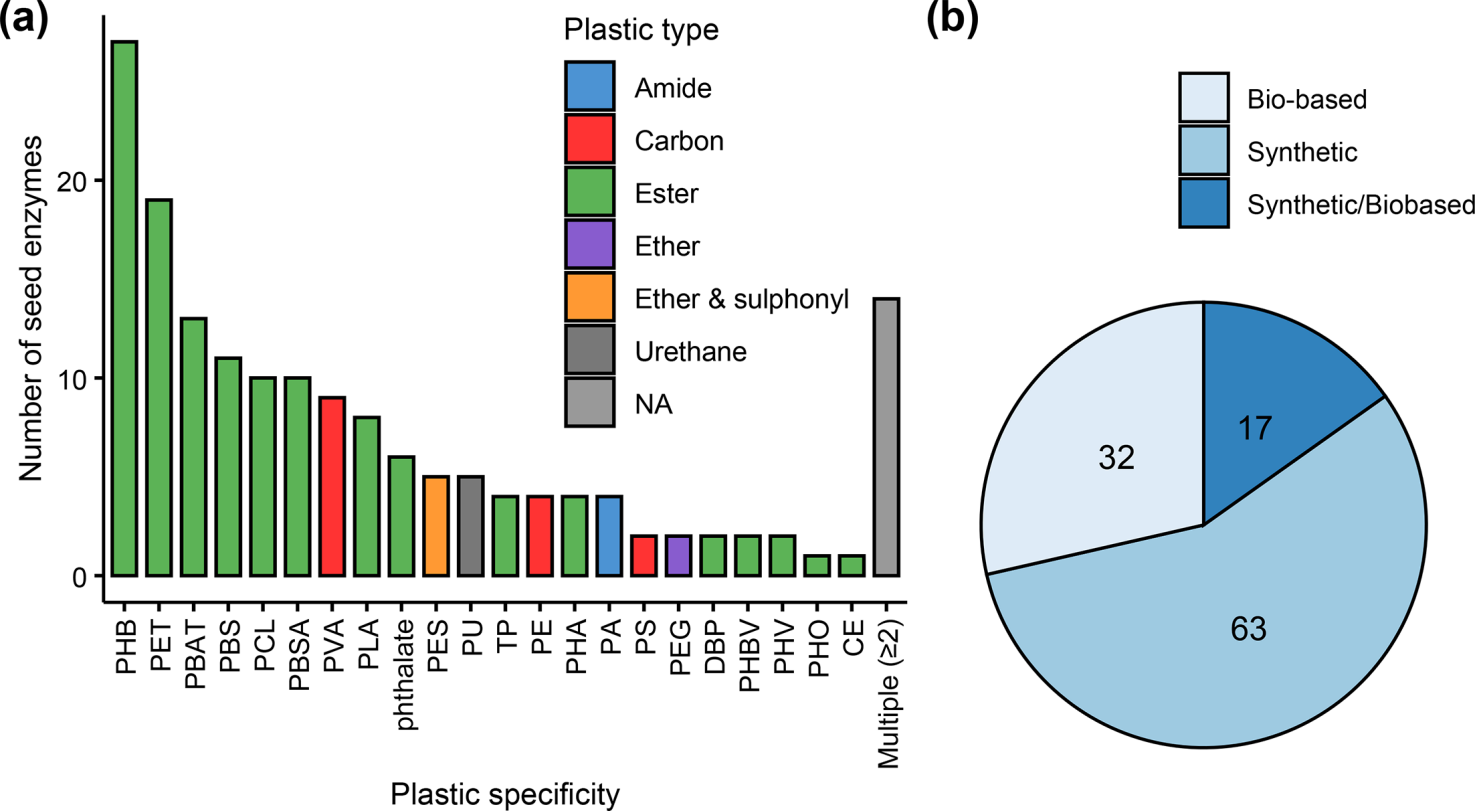
Breakdown of the 112 dereplicated plastic-degrading seed enzymes. (**a**) Number of seed enzyme amino acid sequences per plastic and molecular backbone. Sequences of enzymes capable of degrading multiple (≥2) different plastic types have been included in the counts of each individual plastic, as well as the corresponding ‘Multiple (≥2)’ category. (**b**) Number of seed enzyme sequences per plastic source: bio-based, synthetic or either synthetic or bio-based (‘Synthetic/Bio-based’).

A total of 145 experimentally verified and putative plastic-degrading enzymes were identified in the primary literature, and their amino acid sequences were retrieved. These enzymes were predominantly bacterial, with only nine eukaryotic (fungal) sequences. After dereplication, the 112 plastic-degrading seed enzymes included a high representation of Pseudomonadota (syn. Proteobacteria), Actinomycetota (syn. Actinobacteria) and Bacillota (syn. Firmicutes) (Fig. S3). The genus with the greatest number of plastic-degrading seed enzymes was *Pseudomonas* (12 sequences), followed by *Comamonas*, *Stenotrophomonas* and *Thermobifida* (5 sequences for each genus). Our dataset also included 12 seed enzymes derived from organisms of unresolved taxonomy: 11 from unclassified bacterial taxa and one from an unclassified fungal lineage. Overall, the seed enzymes were mainly annotated as PHB-, PET- and PBAT-degrading proteins with 27, 19 and 13 representatives (Fig. 2). Fourteen of the seed enzymes had published evidence of multi-plastic (i.e. ≥2 targets) degradation capability. No PVC- or PP-degrading enzymes, with available amino acid sequences, were identified in the primary literature. The amino acid sequences of all seed enzymes are available on Zenodo (https://doi.org/10.5281/zenodo.15480170).

### Phylogenetic trees and HMMs

Homologues of the 112 seed enzymes were identified through a blast search of their amino acid sequences against the UniRef100 database and used to infer phylogenetic trees. Either orphan (i.e. from a single seed enzyme) or grouped enzyme trees were constructed, depending on whether there was overlap between the blast hit results of seed enzyme sequences. The resulting 65 phylogenetic trees included 51 orphan and 14 grouped trees. Representative examples of each tree type are included in Fig. S1. A tree could not be built for one PHB-degrading seed enzyme sequence (ID number Q71KW6), since its only blast hit was itself. In contrast, a PEG dehydrogenase from *Sphingopyxis macrogoltabida* (ID A9ZLZ8) had over 400,000 blast hits, although only 12 of these hits had a percent identity of >60% to the target sequence, and 2,205 representatives remained after 70% clustering and filtering to infer the phylogenetic tree. For most other seed enzyme sequences, the number of blast hits ranged from several hundred to several thousand. See Fig. S4 for a summary of the number of blast hits for each plastic type.

Phylogenetic trees were used to establish the HMM input and negative control sequences for HMM screening. In the absence of any experimental evidence, the sequences determined to be ‘positive’ were those within the same monophyletic clade as the seed sequence(s) and therefore presumed to have plastic-degrading abilities. In every case, a conservative approach was taken to define the boundary of the seed sequence’s monophyletic clade, with the selection supported by blast percent identity and e-value scores. The ‘negative’ control sequences were established by setting a maximum e-value cut-off which effectively separated the sequences above this threshold from the positive clade, under the assumption that the sequences most distant from the seed sequence have no plastic-degrading ability. Refer to Fig. S1 for illustrative examples. The positive sequences were clustered at 95% identity and used to generate HMM packages with GraftM. The negative control sequences were used to assess the performance (recall and precision) of the generated HMMs.

A precision and recall analysis was conducted to assess each HMM’s performance and determine its optimal e-value cut-off. HMM precision and recall were calculated from the number of true positive, false positive and false negative hits within the control datasets. Aggregated Precision-Recall (PR) curves for all 65 HMMs are shown in Fig. S5. The majority of HMMs were perfect or near-perfect models, i.e. with an area under the PR curve (PR-AUC) of ~1. Only a subset of the HMMs had a PR-AUC less than 0.9, and in all instances, this was due to an imbalance in the positive and negative populations. The precision and recall values were used to calculate the F1 measure and set the HMM search cut-offs. Twelve HMMs with a precision of <95% were excluded from subsequent analyses, resulting in a final number of 53 HMMs.

### Identification of potential plastic-degrading GTDB taxa

**Fig. 3.**
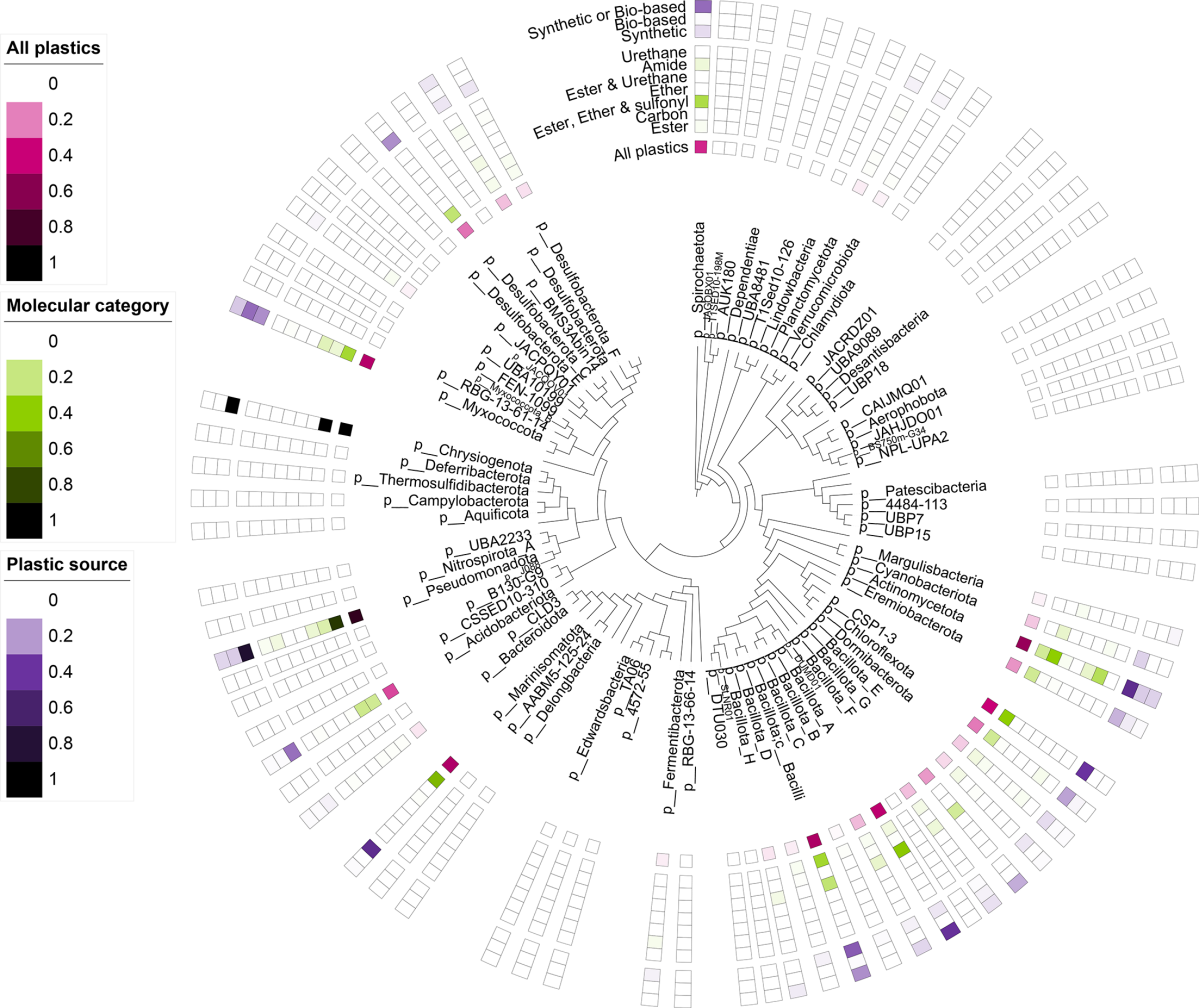
Identification of potential plastic-degrading representatives at the phylum level within the bac120 reference tree of the GTDB (R214) database. The circular heatmaps indicate the proportion of genomes within each clade with plastic-degrading enzyme HMM hits. Target plastic molecular category and source are indicated in different colours alongside a summary of all plastic hits. Tree annotated in iTOL [174].

The benchmarked 53 HMMs (Fig. S6) were used to search GTDB R214 representative genomes for potential plastic-degrading bacterial and archaeal species. Genomes with one or more hits were considered to have potential plastic-degrading capability. Hits were detected in both domains, although substantially more potential plastic-degrading bacterial than archaeal taxa were identified (Figs 3 and S7). The major bacterial phyla containing a high proportion of representatives with hits included Pseudomonadota, Actinomycetota, Bacillota, Bacillota_A, Spirochaetota and Acidobacteriota, with 85%, 55%, 49%, 44%, 34% and 29% of the representative genomes within each clade exhibiting at least one HMM hit, respectively. Potential carbon backbone plastic degraders targeting PE and PVA were most prevalent in Actinomycetota (40% of representatives) and Acidobacteriota (18% of representatives), followed by Pseudomonadota (13% of representatives). The complete taxonomy of all GTDB hits is included in Table S4.

**Fig. 4.**
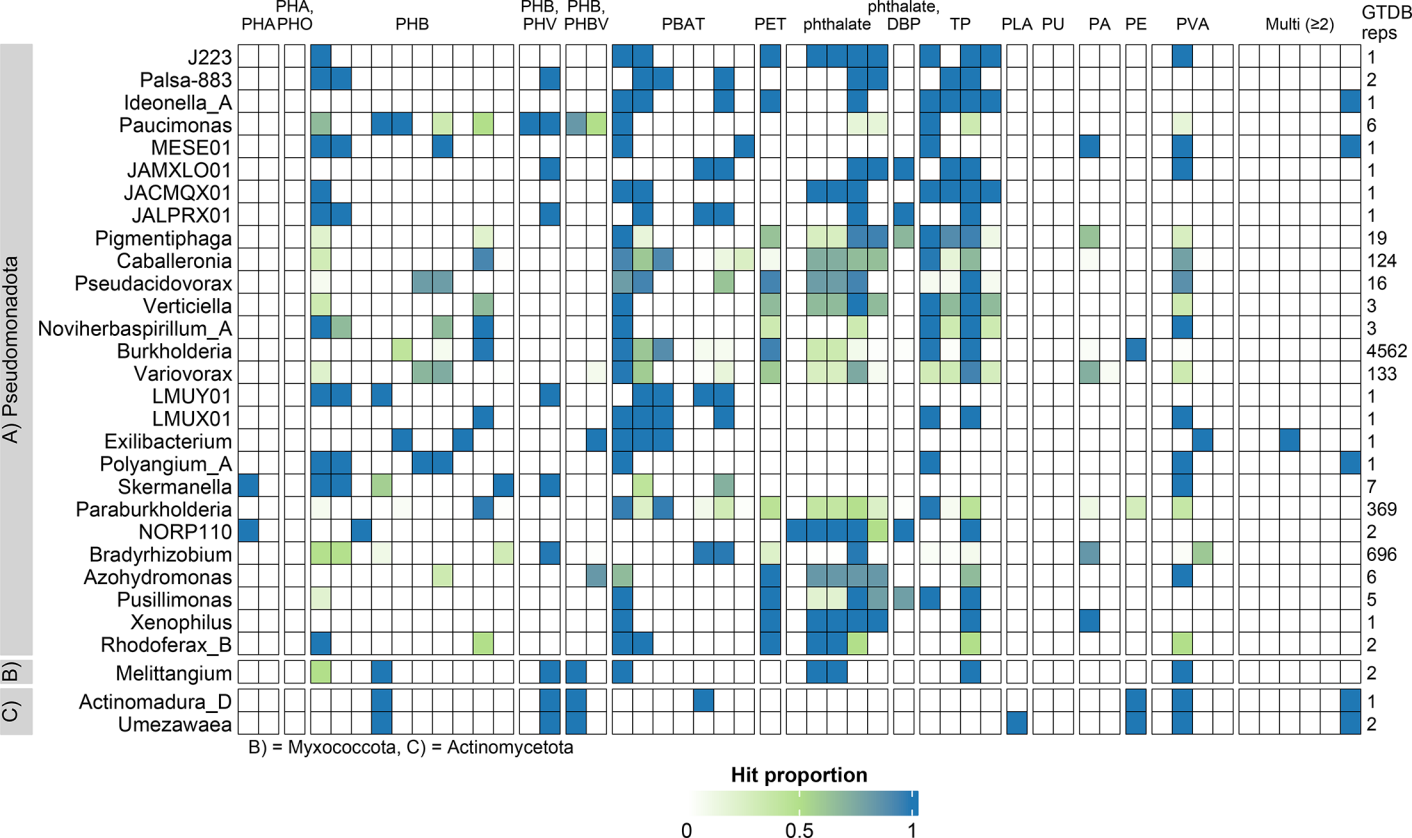
Top 30 putative plastic-degrading genera in the GTDB. Heatmap showing the proportion of GTDB genera representatives with hits for each plastic-degrading enzyme HMM, excluding those with no hits across the entire GTDB. The HMM targets and number of genus representatives are indicated. The genera were selected for the most *multi*-HMM hits rather than the greatest number of hit occurrences, as multiple gene copies and therefore multiple hits of the same plastic-degrading enzyme HMM are possible. Within the multi (≥2) plastic category are HMMs targeting (**i**) PLA, PBS and PBSA; (ii) PLA, PBS, PBSA, PES, PCL and PHB; (iii) PLA, PBS, PBSA, PES and PCL; (iv) PBAT, PBSA, PBS, PES and PCL; (**v**) PLA, PBS, PBSA, PES and PCL; and (vi) PET, PBS, PCL, PA, PBSA, PLA, PHB and PU.

The top GTDB hits are presented in Fig. 4. This heatmap highlights the 30 GTDB genera with the greatest variety of HMM hits (rather than the greatest total number of hits) and includes 27 Pseudomonadota, 2 Myxococcota and 1 Actinomycetota representative genera. One genus, J223, belonging to Burkholderiaceae, had hits for 11 different HMMs encompassing 6 different plastic targets with both ester and carbon backbones. Other notable mentions include *Ideonella_A* with species *P. sakaiensis* (syn. *I. sakaiensis*), a well-known PET-degrader [21]; *Paucimonas*, which includes PHB- and PHV-degrader *Paucimonas lemoignei* [63]; *Burkholderia*, containing phthalate-degrader *Burkholderia cepacia DBO1* [98]; and *Polyangium_A* (reclassified as *Caldimonas* in GTDB R226) with *Caldimonas brevitalea* (syn. *Polyangium brachysporum*), a demonstrated PET-degrader [40]. All of these genera included HMM hits consistent with the published evidence of their plastic-degrading ability, plus additional targets.

Within Archaea, 4 of the 21 phyla had plastic-degrading gene hits. However, three of these lineages only had hits within <10% of their representative species genomes. The fourth phylum, Asgardarchaeota, had hits in 18% of its representatives (Fig. S7).

### Identification of potential plastic-degrading enzymes in environmental metagenomic samples

**Fig. 5.**
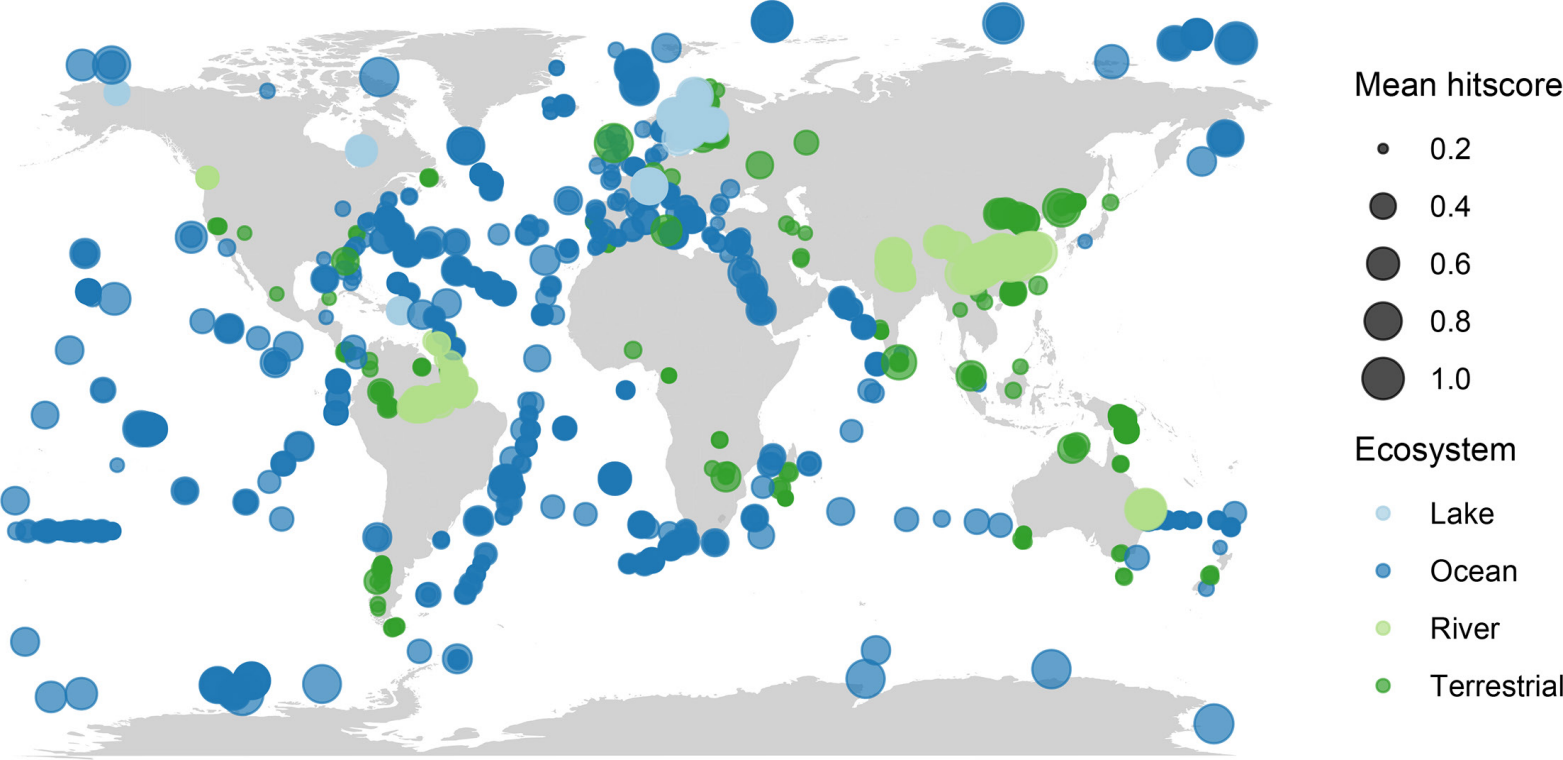
Detection of potential plastic-degrading enzymes on a global scale. Shown are the locations and mean normalized hit scores for all plastic-degrading enzyme HMMs at each metagenome sampling site included in this study. There were no sites with a mean normalized hit score less than 0.2.

**Fig. 6.**
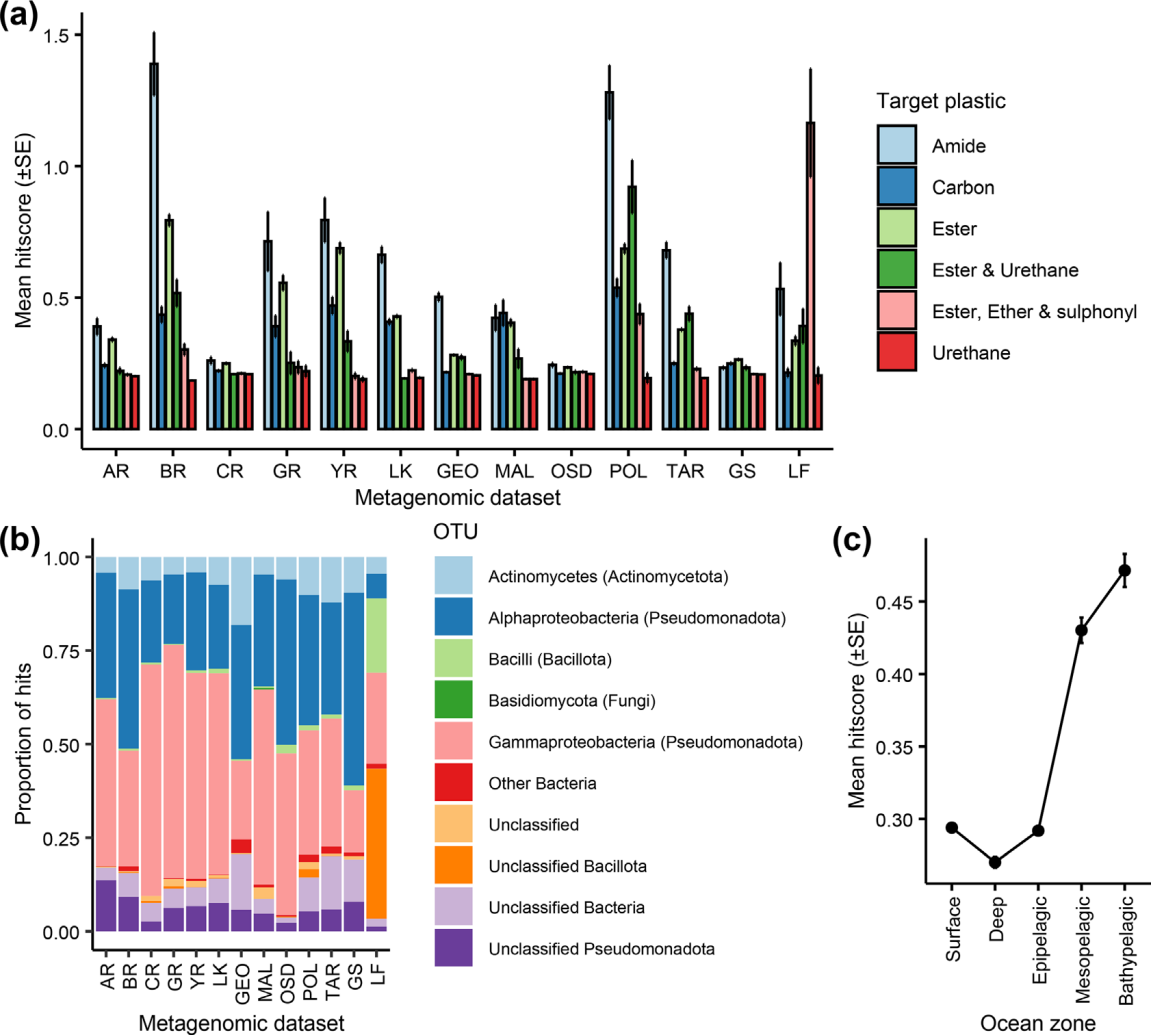
Quantification of potential plastic-degrading enzymes across datasets, plastic types, prokaryotic lineages and ocean zones. (**a**) Normalized plastic-degrading enzyme HMM hit scores from the Amazon River (AR), Brisbane River (BR), Canadian river system (CR), Ganges River (GR), Yangtze River (YR), Lakes (LK), GEOTRACES (GEO), Malaspina (MAL), 2014 Ocean Sampling Day (OSD), Polar Oceans (POL), Tara Oceans (TAR), Global Soil (GS) and Landfill (LF) metagenomes, differentiated by target plastic type. Note: multiple listed types (e.g. ‘Ester, Ether and sulphonyl’) are indicative of HMM(s) targeting genes encoding multi-plastic-degrading enzymes. (b) Taxonomic profile of plastic-degrading enzyme HMM hits within the metagenomic datasets as assigned by GraftM. Note: the NCBI taxonomy output of GraftM has been converted to the GTDB equivalent. The breakdown is by class except for unclassified microorganisms and bacteria with low (<2%) hits across all metagenomes. The ‘Other Bacteria’ group includes Acidimicrobiia (Actinomycetota), Clostridia (Bacillota), Deinococci (Deinococcota), Gemmatimonadetes (Gemmatimonadota), Spirochaetia (Spirochaetota), unclassified Actinomycetota and unclassified Bdellovibrionota. (**c**) Mean normalized HMM hit scores for metagenome samples within each vertical ocean zone: 0–20 m (surface), 20–50 m (deep), 50–200 m (epipelagic), 200–1,000 m (mesopelagic) and >1,000 m (bathypelagic).

Metagenomic samples from across the world’s oceans, rivers and lakes, together with global soil and landfill leachate samples, were screened for genes encoding potential plastic-degrading enzymes. See Fig. 5 for the geographic distribution of plastic-degrading gene hits, while Fig. 6(a) shows the normalized hit scores by metagenome. Overall, the Brisbane River, Yangtze River, Lakes and Polar Oceans samples had a greater abundance of hits, followed by the Amazon River, Tara Oceans, Global Soil and Landfill samples. In comparison, there were very few hits within the Canadian river system, Global Soil Microbiome and 2014 Ocean Sampling Day datasets.

As shown in Fig. 6(a), ester-, ether- and sulphonyl-degrading enzyme HMMs had the greatest mean hit scores localized in the landfill leachate samples [1.16±0.21 standard error (SE)], while amide-degrading genes were moderately abundant across most metagenomes (mean hit score 0.52±0.01 SE). Ester-degrading genes were common in both freshwater, Malaspina Deep Ocean and Polar Ocean samples. Urethane-degrading enzyme HMMs had low mean hit scores across all metagenomes. Surprisingly, HMMs targeting genes for the degradation of synthetic plastics with a strict carbon backbone, including PE and PVA, showed a considerable mean hit score in some rivers, Malaspina Deep Ocean samples and a peak at the Polar Oceans samples (Fig. 6a). Among these hits, putative PE-degrading genes were most abundant in the Polar Oceans, Malaspina and Ganges River samples, whereas PVA-degrading genes predominated the carbon-degrading hits from the Brisbane River (Fig. S8).

The taxonomic profiles of the hit sequences were generally similar across the different aquatic metagenomes (Fig. 6b) with a high representation of Pseudomonadota, including Actinomycetia, Alphaproteobacteria and Gammaproteobacteria. While also present in the soil and landfill samples, the proportions differed substantially. Approximately 60% of the landfill hits were classified as Bacillota sequences (~20% Bacilli and ~40% unclassified Bacillota), which represented <3% of HMM hits across all other metagenomes. A relatively large proportion (up to 15%) of HMM hits could not be classified beyond domain. There were only three eukaryotic hits identified across the entire dataset. These were identified as Exobasidiales (Basidiomycota) and occurred within the Malaspina Deep Oceans samples (Fig. 6b). Although typically associated with plant hosts, this fungal lineage has been found in deep-sea environments such as the Mariana Trench [137].

Within the ocean metagenomes, there was a correlation between the sampling depth and the mean hit scores. The highest average HMM hit scores occurred within the deepest vertical ocean zones: the mesopelagic (200–1,000 m deep) and bathypelagic (>1,000 m) layers (Fig. 6c).

## Discussion

Plastic pollution has become a ubiquitous component of many environments across the globe. However, relatively few microorganisms capable of plastic waste biodegradation have been isolated and identified to date. This is primarily due to practical challenges, including the inability to culture most microbes. Metagenomic sequencing of environmental samples paired with bioinformatic analysis can be an effective way to overcome these challenges and to directly explore the diversity of plastic-degrading enzymes. Apart from the study by Zrimec *et al*. [39], there have been few bioinformatics-based assessments of potential plastic-degrading microorganisms in the environment. Here, we used the amino acid sequences of enzymes with known or predicted plastic-degrading function to successfully generate a set of HMMs, which were subsequently screened using positive and negative control datasets. A customized search cut-off was established for each HMM which optimized model performance in terms of precision and recall. The final 53 non-redundant plastic-degrading enzyme HMMs spanned 20 different plastics and included amino acid sequences from both bacterial and eukaryotic microorganisms.

While the HMMs were built from predominantly Pseudomonadota (synonym Proteobacteria), Actinomycetota (Actinomycetia) and Bacilli (Firmicutes) species, HMM hits occurred across a diverse range of bacterial lineages within the GTDB, as seen in previous studies [38, 138]. However, Pseudomonadota had the greatest proportion of representatives with plastic-degrading HMM hits. This result is supported by multiple previous reports of encoded and experimentally verified plastic-degrading enzymes associated with members of this phylum [e.g. 54, 56, 65, 67, 138]. A recent predictive study also concluded that Pseudomonadota has the greatest abundance of putative plastic-degrading proteins across all polymer types [139]. The community profiles of environmental metagenome hits were generally consistent with the distribution of hits across GTDB phyla. The most dominant phyla represented were Pseudomonadota (including the classes Alphaproteobacteria, Gammaproteobacteria and unclassified Pseudomonadota), Actinomycetota (Actinomycetes), Bacillota (Bacilli) and Bacteroidota (Bacteroidia). Alphaproteobacteria, Gammaproteobacteria, Bacteroidia, Actinomycetes and Bacilli taxa are all associated with plastic particle biofilms [140]. There was some variation in the community profiles of HMM hits between metagenomic datasets, but the dominant classes were present in all ecosystems. Most notably, Bacilli hits were much more abundant in the landfill samples than any other dataset. Bacilli is one of the most common bacterial classes found in landfill samples, likely due to the ability of members of this class to degrade natural polymers such as cellulose and lignin, that are abundant components of organic waste in landfills [141, 142]. Many other metagenome hits (1–15%) could not be classified beyond domain (Fig. 6), suggesting that unknown microorganisms may also hold key solutions for breaking down plastic pollution and supporting environmental sustainability.

Screening the GTDB identified the top 30 genera with the greatest diversity of genes encoding putative plastic-degrading enzymes. This list included well-known plastic degraders such as *P. sakaiensis*, and taxa not currently known to possess plastic-degrading abilities, such as Pseudomonadota genera J223, Palsa-883 and MESE01 (Fig. 4). Interestingly, the genera with published evidence of plastic degradation had hits consistent with our knowledge of their plastic targets, plus hits for additional targets. In the case of PET-degrading *P. sakaiensis* [21], hits occurred for PET, PBAT, phthalate, TP (all esters) and a multi-plastic-degrading enzyme HMM targeting PET, PBS, PCL, PA, PBSA, PLA, PHB and PU (ester or urethane backbones) (Fig. 4). It is known that plastic-degrading enzymes can act on multiple targets [36, 101, 103], or a bacterium may produce multiple different enzymes that act in concert to metabolize a plastic substrate [20, 25, 58, 63, 66, 98]. Experimental validation is required to confirm the predicted plastic-degrading ability of the identified genera. Regardless, our list of genera (Fig. 4) provides a set of strong candidates warranting further investigation.

The presence of plastic-degrading enzyme HMM hits within Archaea was an unexpected finding. No plastic-degrading archaeal taxa have been described in the literature, although archaeal colonies have been observed growing on the surface of plastic articles found in the ocean [143, 144]. The most archaeal hits were within Asgardarchaeota. This phylum is thought to contain the closest prokaryotic relatives to Eukarya [145], and some members such as the Helarchaeia, Hermodarchaeia, Thorarchaeia and Lokiarchaeia were inferred to have the potential to activate and anaerobically oxidize short-chain hydrocarbons, most likely alkanes [146, 147]. Alkane degradation has been proposed to be involved in PE degradation, since *n-*alkanes share structural similarities with PE [148, 149]. The GTDB Asgardarchaeota hits included predominantly Heimdallarchaeia and Lokiarchaeia, including Helarchaeales (syn. Candidatus Helarchaeota). A comparison of the seed enzyme amino acid sequences of HMMs with Asgardarchaeota hits and two published Helarchaeota MAGs containing identified alkane degradation enzymes [147] yielded hits with 27.0% (Hel_GB_A MAG) and 25.7% (Hel_GB_B MAG) identity to an ester-, ether- and sulphonyl-degrading seed enzyme sequence (ID A4UZ14). Therefore, it is possible that the HMMs have identified true hits for Asgardarchaeota taxa capable of enzymatic degradation of these plastics. The essential next step will be to validate these predicted plastic-degrading capabilities using transcriptomic or culture-based approaches to confirm their functional relevance.

Since the molecular backbone of PE closely resembles that of alkanes, it has been proposed that the metabolic pathways and enzymes involved in PE biodegradation are similar to those used for alkane degradation [148, 150]. Due to the requirement of functional evidence of primary plastic material digestion (see *Construction of HMMs* in *Methods* section), we did not include published alkane-degrading enzymes in our analysis unless there was also evidence of polymer biodegradation. However, recent studies suggest that alkane-degrading enzymes may also contribute to plastic degradation, with alkane monooxygenase and alcohol dehydrogenase implicated in PS degradation [151], and alkane hydroxylation and alcohol dehydrogenation proposed as key steps in PE degradation [152]. We recommend considering alkane-degrading enzymes such as these in future *in silico* detection approaches.

Rivers play a key role in transporting plastic pollution from terrestrial sources into the ocean ecosystem [153]. Our results indicate that enzymatic biodegradation may also occur within the river channel, since plastic-degrading enzyme hits were abundant in the highly polluted [153] Ganges and Yangtze Rivers, as well as the Brisbane River. Lakes may also function as a reservoir of accumulated plastic debris and plastic-degrading microorganisms, as evidenced by the high plastic-degrading enzyme HMM hit scores of samples from lakes such as Lake Odrolstjärnen, Lake Nästjärnen and Lake Haukijärvi (Fig. S9). Indeed, plastic leachate has been observed to be a readily available source of carbon for microbial communities in lake ecosystems [154]. Across ocean metagenomes, the distribution of putative plastic-degrading genes was highly variable (Fig. 6a). The Polar Ocean samples had the greatest mean normalized hit scores followed by the Malaspina Deep Ocean samples, while the primarily shallow and coastal Ocean Sampling Day samples had very few hits. The mean hit scores increased with ocean depth, a trend also observed by Zrimec *et al*. [39]. Despite the distance from sources of anthropogenic influence, microplastics have been discovered in deep ocean sediments [8], the Antarctic marine system [155] and the North-East Atlantic Ocean [156]. Modelling also predicts that deep-ocean environments act as a sink for microplastic pollution [157, 158]. Furthermore, multiple microorganisms capable of degrading plastics, including *Alcanivorax*, *Pseudomonas* (both Pseudomonadota) and *Rhodococcus* (Actinomycetota) species, have been isolated from cold, deep-sea environments [159, 160]. A possible explanation for the elevated abundance of plastic-degrading gene hits in these extreme ocean environments is that plastics reaching these deep ocean or polar regions have already experienced extensive weathering, including UV degradation, chemical oxidation and physical fragmentation, making them more accessible to biodegradation.

This study included a wide range of plastic types, ranging from PHA, a natural polymer that functions as a carbon storage compound in bacteria [161], to synthetic polymers consisting of a sole carbon backbone, such as PE, PS and PVA (Table 1). PVA was generally considered to be a biodegradable polymer, since it is water-soluble, but it has become a major pollutant of industrial wastewater [162]. Enzymatic degradation by bacterial genera such as *Pseudomonas* and *Spingomonas* could offer a solution [163]. Hits for genes of PE-depolymerizing enzymes were low in most metagenomes, but present in the Ganges River, Malaspina Deep Ocean and Polar Oceans samples. Enzymes with the potential to depolymerize non-hydrolysable synthetic plastics such as PE could have evolved over the last few decades since their invention, and it has been argued that new enzymatic functions can evolve in such timeframes [161]. However, it seems more likely that such enzymes evolved over longer periods, not to break down plastic, but to target natural molecules, e.g. complex biopolymers such as cellulose, chitosan and alginate [164–166], and that their plastic-degrading potential has only been recognized in recent years. This enzymatic arsenal for biopolymers could enable microorganisms to depolymerize oxidized synthetic polymers of similar composition. A recent study [139] argues that plastic-degrading potential is nearly universal among prokaryotes, with environmentally shaped enzyme repertoires, rather than restricted to a small set of rare specialists. In either case, this potential can be further enhanced by directed evolution, a powerful application of artificial selection, that offers a robust framework for accelerating protein diversification and studying the evolution of plastic-degrading enzymes [167]. This has recently been demonstrated with the improvement of the catalytic activity and thermostability of a PET-degrading enzyme [168]. Innovations in this area are essential to enable the application of plastic-degrading enzymes on an industrial scale [169].

HMMs are effective for efficiently screening large datasets; however, this study focuses on identifying putative rather than experimentally confirmed plastic-degrading enzymes. While a conservative approach was applied to construct the HMMs and determine the most appropriate cut-off for every individual model by maximizing its precision and recall, the methods themselves are heuristic in nature. Conversely, the stringent thresholds may have caused true plastic-degrading enzymes to be overlooked. The application of a 70% identity clustering threshold for datasets of >5,000 sequence homologues during the construction of the HMMs may have impacted HMM sensitivity by excluding distant yet functionally relevant homologues from the resulting HMM profiles. However, our approach still encompasses a more diverse array of plastic targets than existing methodologies. For example, PlasticEnz integrates HMMs with machine learning to improve predictive performance, yet our models span more plastic types (20 versus 11) and achieved higher overall F1 scores during benchmarking (0.82–1.0 across all models compared with 0.86 and 0.58 for PlasticEnz PET and PHB models, respectively, using default cut-offs) [170], although the evaluation datasets differ. Similarly, PEZy-miner reports comparable F1 scores but is restricted to 11 plastic types [171]. Although it also constructs models from blast-derived homologues, these are capped at 5,000 sequences [171], whereas our clustering approach incorporates larger datasets and captures greater sequence variability. Furthermore, neither PlasticEnz nor PEZy-miner accounts for the phylogenetic distribution of experimentally verified seed sequences during model construction, an aspect explicitly considered in our workflow. Finally, while KEGG [172] is a standard resource for metabolic pathway identification, it includes only a narrow selection of major plastic types and largely focuses on pathways for monomers or downstream breakdown products, rather than the primary polymer degradation steps that our HMMs aim to detect.

Ultimately, the value of this bioinformatics-based approach is to guide experimental research towards the best chance of successful and informative findings, which, in this case, is the discovery of novel plastic-degrading enzymes. To this effect, it is recommended to focus future research efforts on experimentally verifying the plastic-degrading potential of promising lineages identified in our study, such as the genera highlighted in Fig. 4 and microorganisms located in deep ocean, polar ocean and polluted river ecosystems. This could be achieved through heterologous expression and activity assessment of the genes identified in this study, or sample enrichment cultures, eventually leading to isolates of promising microbial strains. Obtaining genome sequences of isolates will allow a careful annotation of genes encoding enzymes with plastic-degrading potential. This step is important for a robust enzymatic *in silico* characterization, since sequence-based protein modelling of all final seed enzymes used in our study (Tables S5 and S6) suggests that some sequences are not complete. For example, in some cases, only the alpha-helix was reported (Table S5), hampering predictions of potential binding pockets and three-dimensional structures. For other enzyme sequences, such as those targeting PHB and PHBV, the current machine learning-derived models provided by AlphaFold [173] appear to be inaccurate or incomplete. They predict long C-terminal tails with an unusual shape, a structure that might be difficult to maintain in nature (Table S5). More complete sequences and better model predictions will help, but ultimately, the capabilities of these enzymes must be validated through laboratory experiments. Advancing this research might be tedious and costly, but potential rewards are high. The identification of effective plastic-degrading enzymes, especially those targeting common synthetic polymers, could be a key contribution to solving the world’s plastic pollution crisis.

## Supplementary material

10.1099/mgen.0.001814 Supplementary Material 1.

10.1099/mgen.0.001814 Supplementary Material 2.

10.1099/mgen.0.001814 Supplementary Material 3.

## Abbreviations

DBP: dibutyl phthalate
GTDB: Genome Taxonomy Database
HMMs: Hidden Markov Models
MAG: Metagenome-assembled genome
MO: microorganism
PAZy: plastic-active enzymes
PE: polyethylene
PET: polyethylene terephthalate
PHAs: polyhydroxyalkanoates
PHB: polyhydroxybutyrate
PLA: polylactic acid
PP: polypropylene
PR: Precision-Recall
PR-AUC: area under the PR curve
PS: polystyrene
PVC: polyvinyl chloride
SE: standard error
