# Supplementary material for: Predicting the plastic biodegradation potential within microbial lineages and across global ecosystems
Source: Microb Genom. 2026 Aug 11;12(8):001814. doi: 10.1099/mgen.0.001814 (PMC13461043; doi:10.1099/mgen.0.001814)
Supplement: Supplementary file 1 [file mgen-12-01814-s001.pdf]

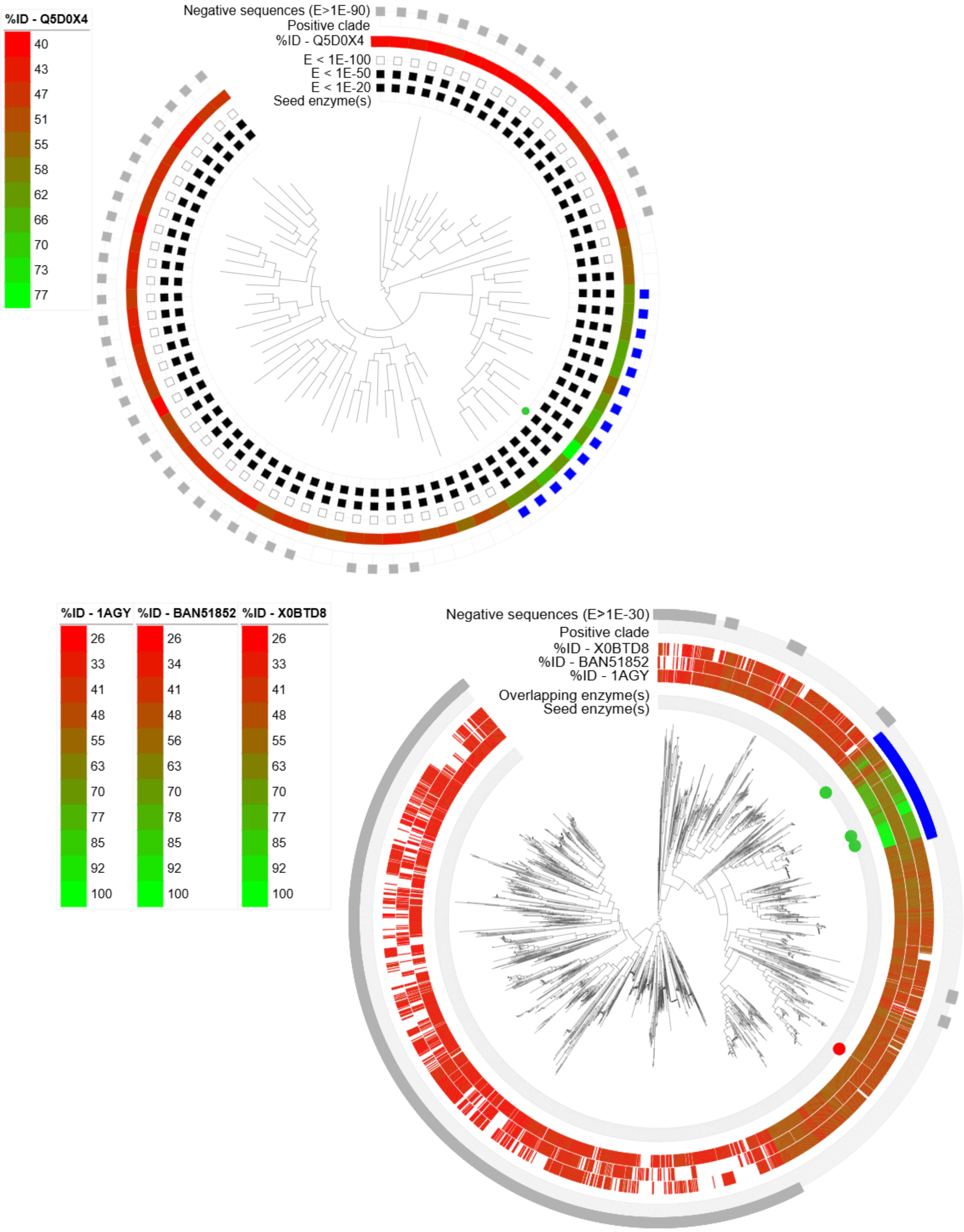


**Supplementary Figure S1.** Representative examples of protein sequence phylogenetic trees inferred from the plastic-degrading seed enzyme amino acid sequence(s) and their BLAST hits. The manually selected "positive" clade of sequences is indicated in blue, and the "negative" sequences which had a BLAST e-value above the determined cut-off are shown in grey. The upper tree is an example of an orphan enzyme's (i.e., a seed enzyme which did not have any other seed enzyme amino acid sequences within its BLAST hits) phylogenetic tree,. The lower tree is an example of a grouped tree with 3 combined seed enzyme sequences (green dots) and their homologues, with an additional overlapping seed enzyme sequence (red dot) that was excluded from the group and treated as an orphan.

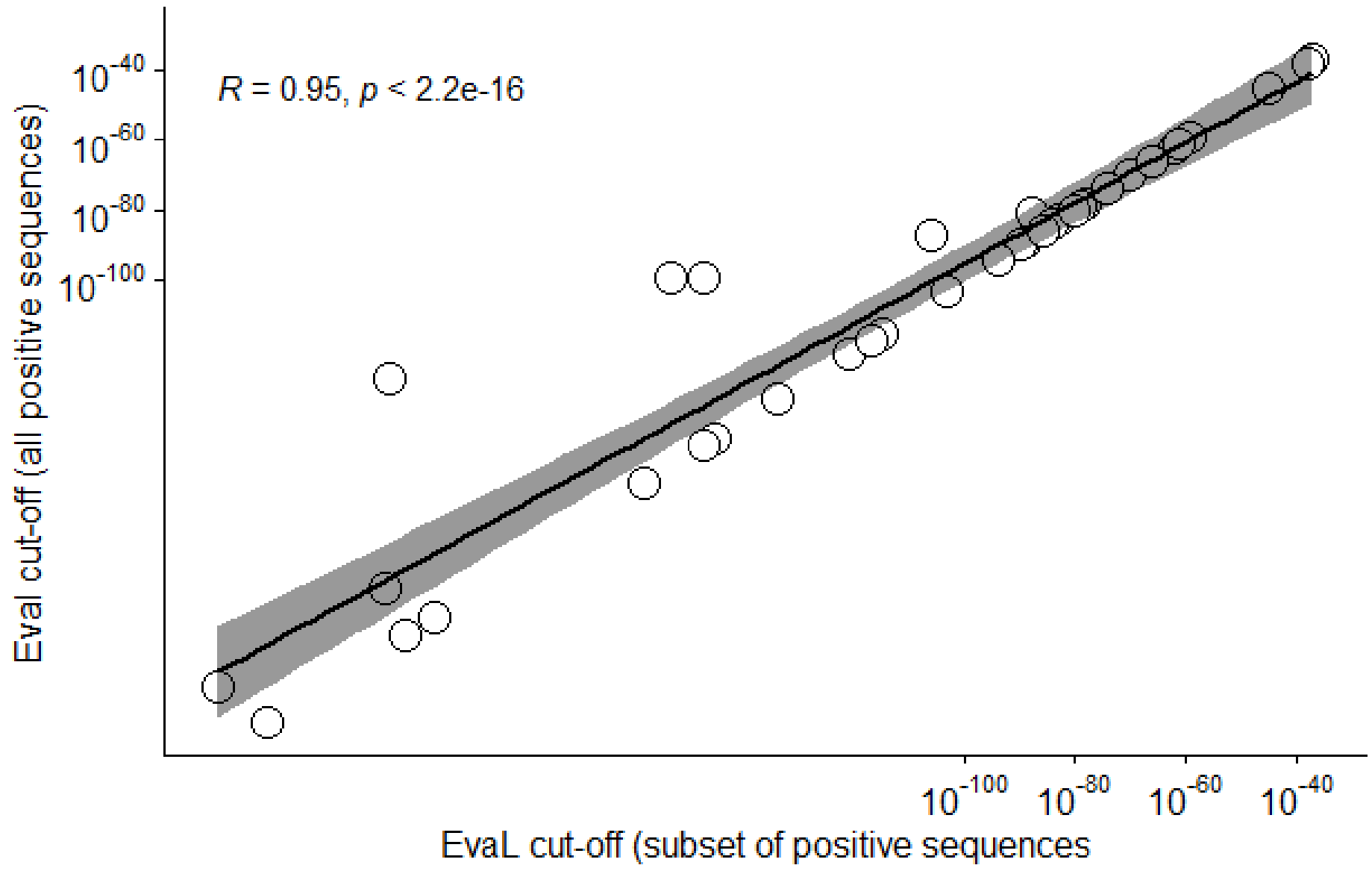


**Supplementary Figure S2.** Influence of positive control sequence dataset on determination of optimal e-value threshold for the set of plastic-degrading enzyme HMMs. Either all positive sequences (y axis) or the positive sequences excluded from HMM generation during pre-clustering (x axis) were screened as a positive control dataset alongside the negative control dataset. The optimal e-value threshold was determined from maximum F1 score. Pearson's correlation coefficient was 0.95, indicating that either method would provide a similar outcome.

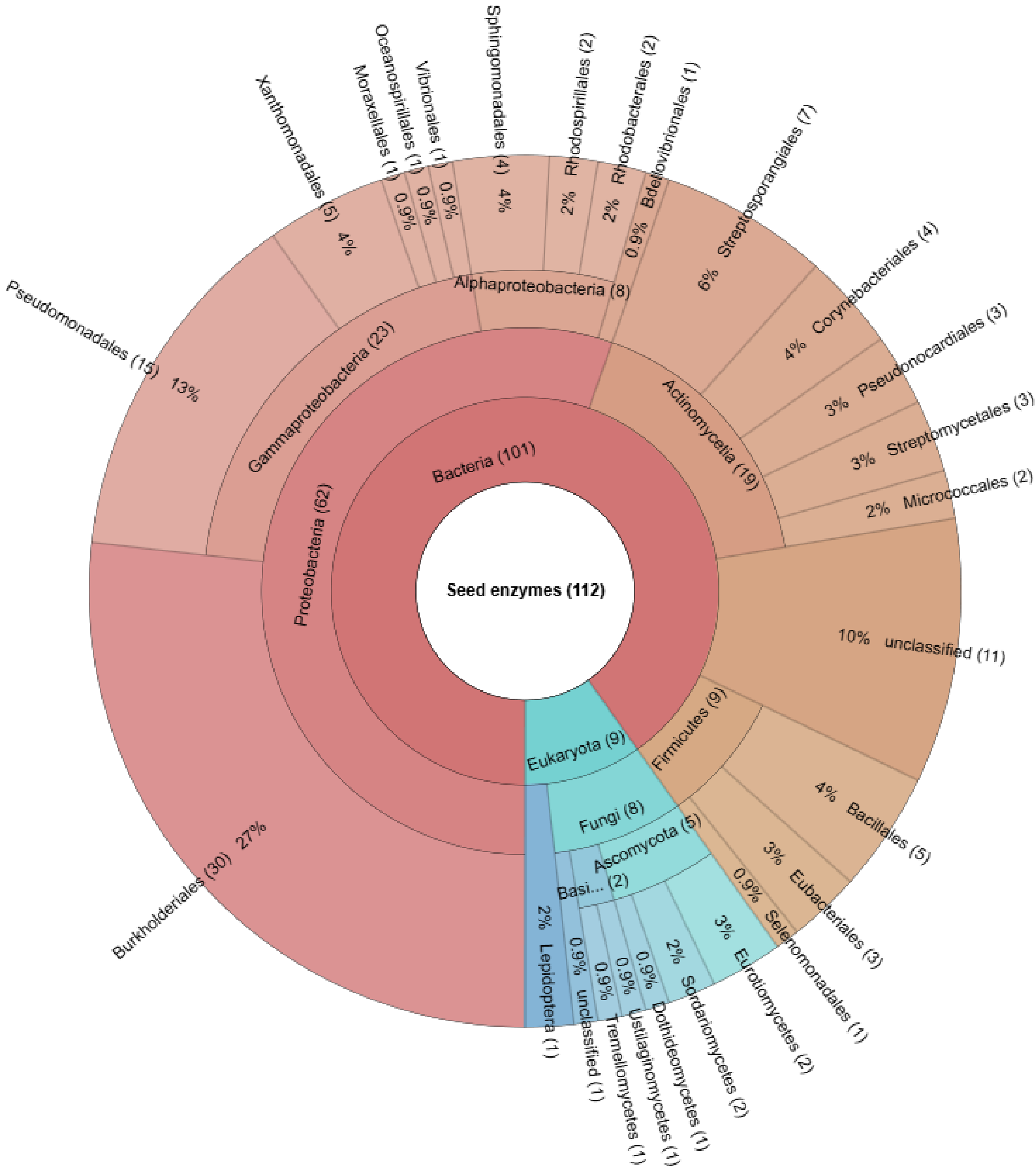


**Supplementary Figure S3.** Krona plot showing taxonomic breakdown of the 112 dereplicated plastic-degrading seed enzymes. The number of seed enzymes for each classification is indicated in brackets.

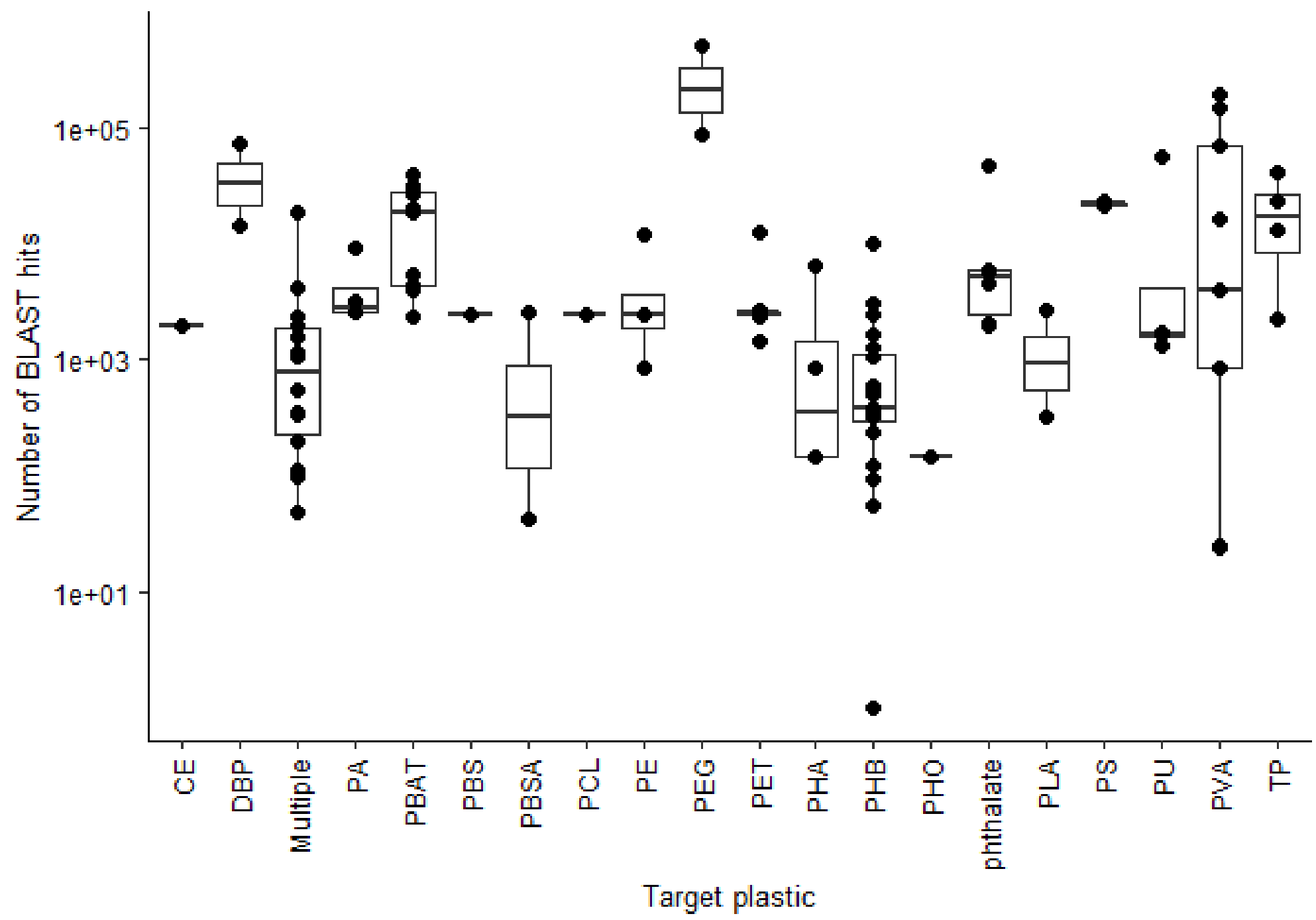


**Supplementary Figure S4.** Summary of UniRef100 BLAST hits for the 112 dereplicated seed enzyme amino acid sequences, differentiated by target plastic specificity. Sequences of seed enzymes capable of degrading ≥2 different target plastics are included in the "Multiple" category.

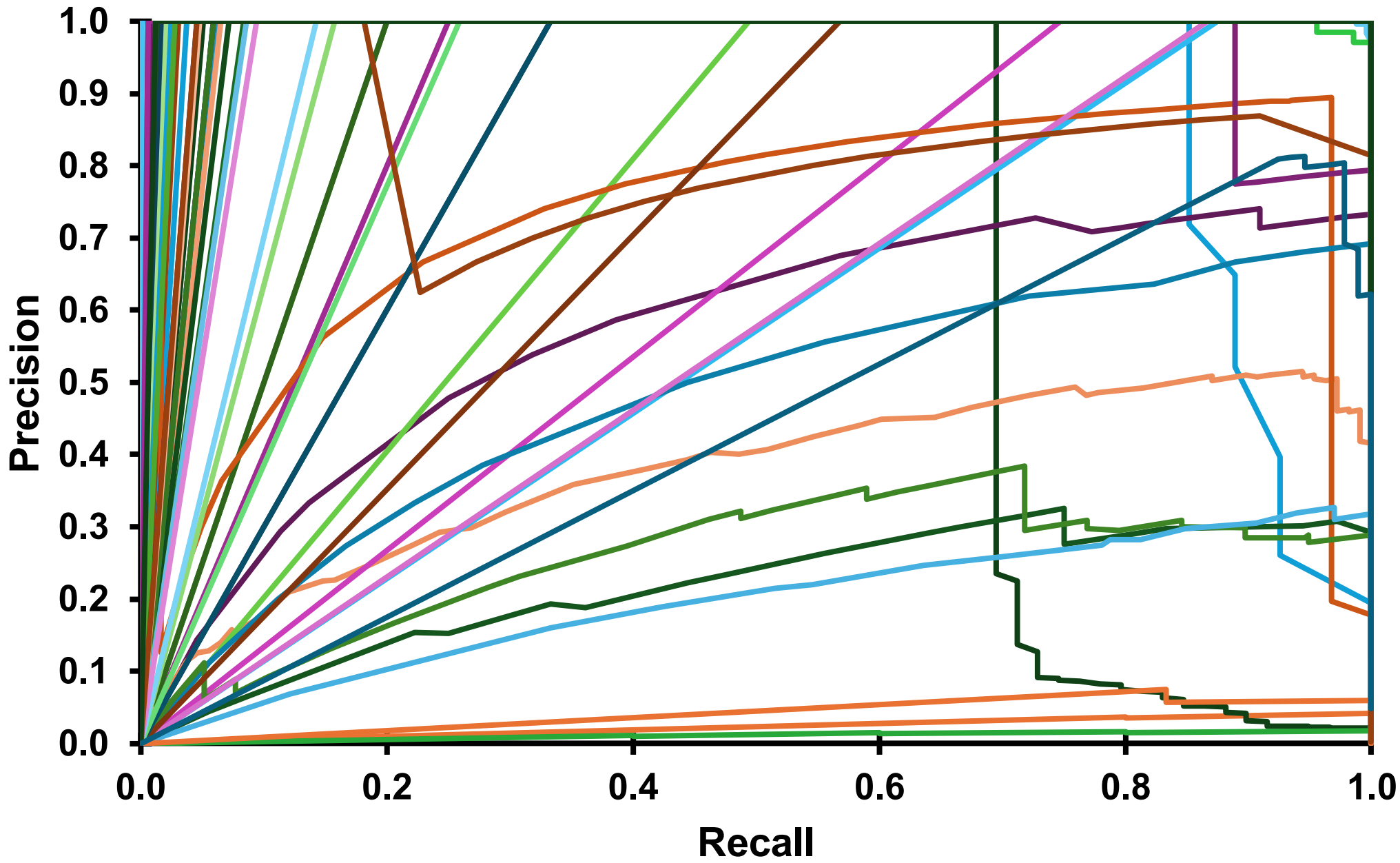


**Supplementary Figure S5.** Aggregated Precision-Recall curves for all 65 plastic-degrading enzyme HMMs showing the trade-off between precision and recall when ranging the HMM e-value search cut-off. The majority of HMMs have perfect or near-perfect models with precision and recall both reaching 1.0.

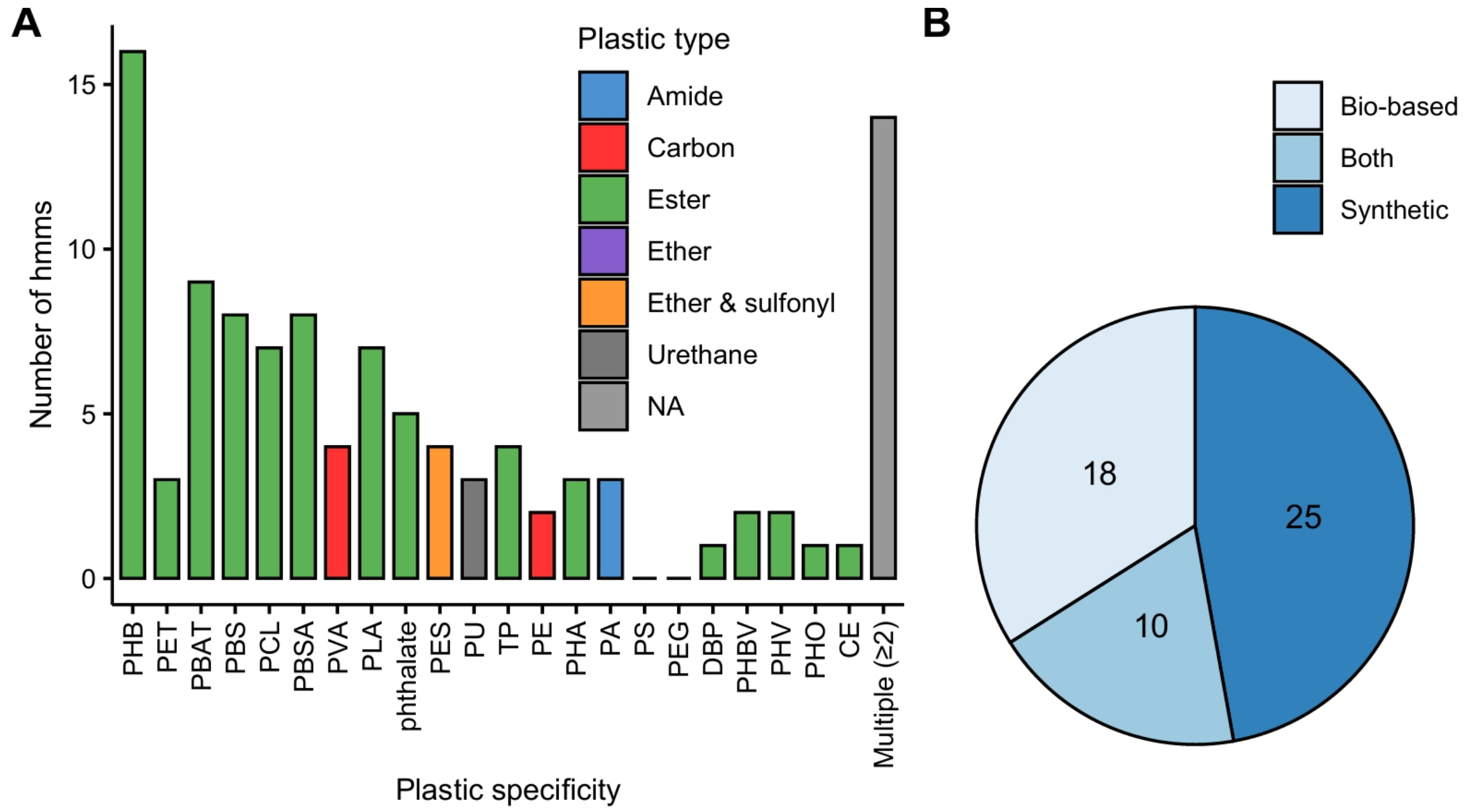


**Supplementary Figure S6. Breakdown of the final 53 plastic-degrading enzyme HMMs. A)** Number of HMMs per target plastic and molecular backbone. HMMs targeting multiple (≥2) different plastic types have been included in the counts of each individual plastic as well as the corresponding "Multiple (≥2)" category. **B)** Number of HMMs per plastic source; Bio-based, Synthetic or either Synthetic or Bio-based ("Both").

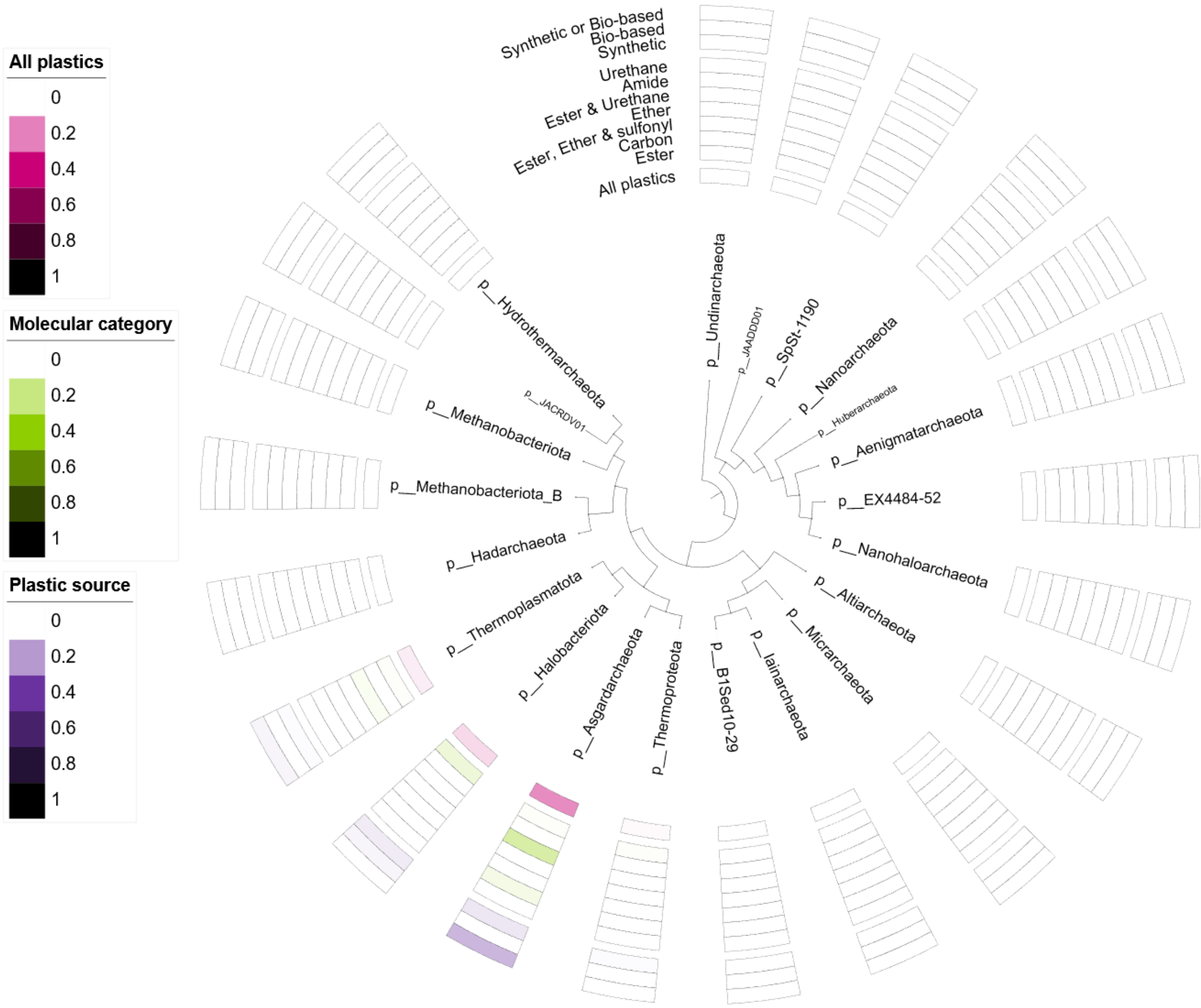


**Supplementary Figure S7.** Identification of potential plastic-degrading representatives at phylum level within the archaeal GTDB (R214) database. The circular heatmaps indicate the proportion of genomes within each clade with plastic-degrading enzyme HMM hits. Target plastic molecular category and source are indicated in different colours alongside a summary of all plastic hits. Tree annotated in iTOL (Letunic & Bork, 2007).

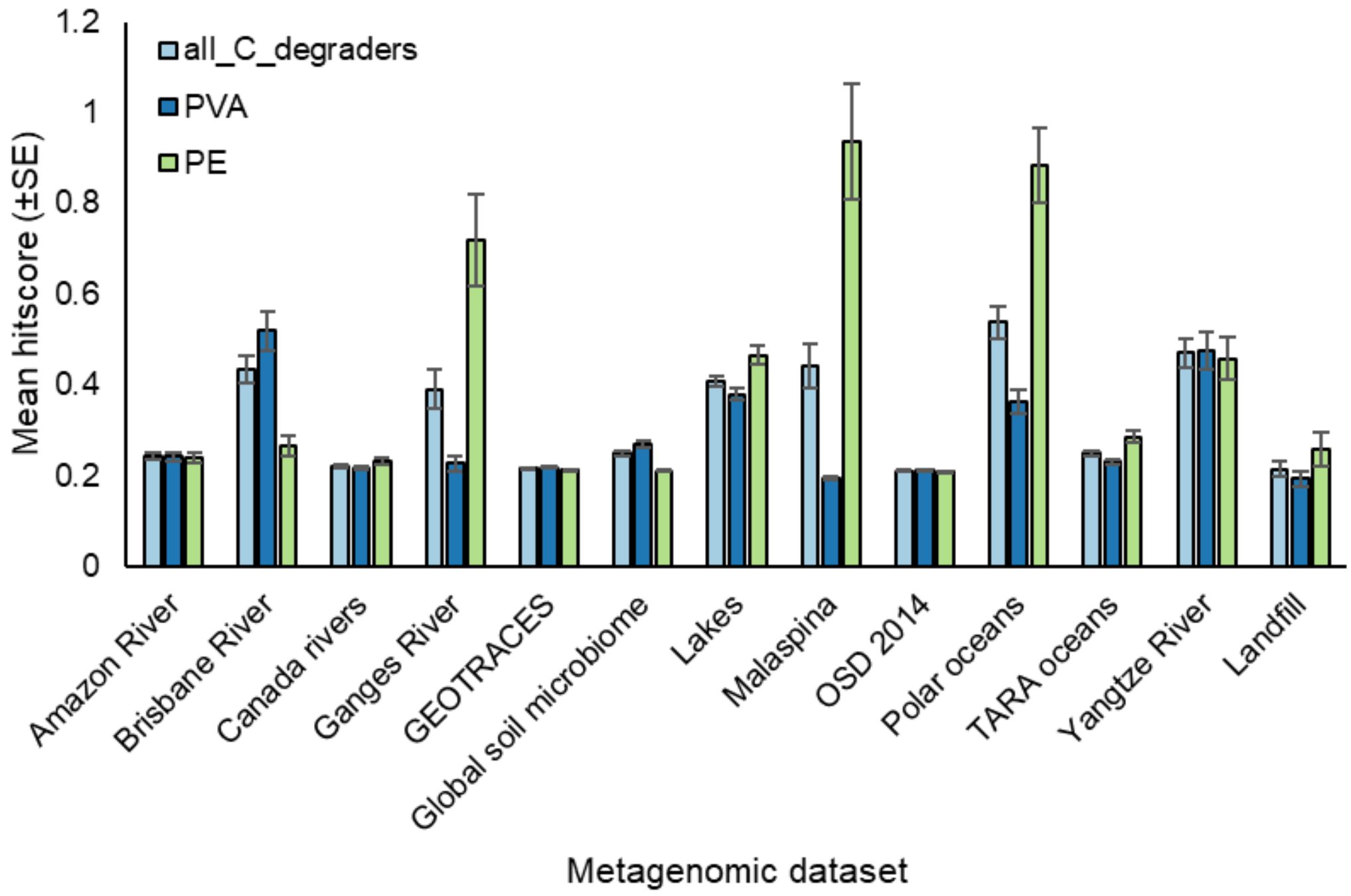


**Supplementary Figure S8.** Mean hit scores across metagenomes for HMMs targeting plastics with a wholly carbon polymer backbone, i.e., PVA and PE. Mean hit scores for PVA and PE degrading enzymes are also shown individually.

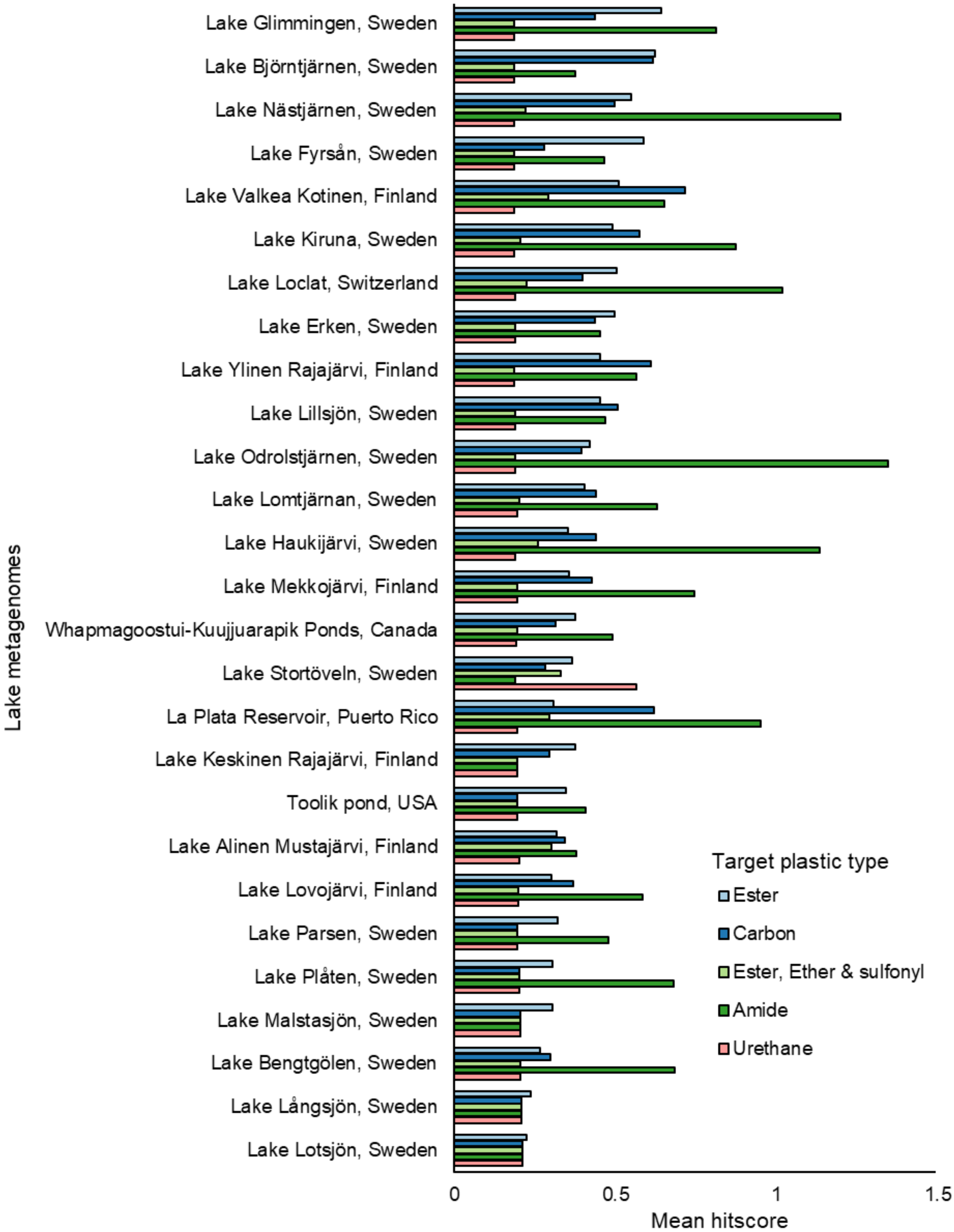


**Supplementary Figure S9.** Mean plastic-degrading enzyme HMM hit scores for the individual lake sampling sites and for each target plastic type.
