# Supplementary material for: Predicting the plastic biodegradation potential within microbial lineages and across global ecosystems
Source: Microb Genom. 2026 Aug 11;12(8):001814. doi: 10.1099/mgen.0.001814 (PMC13461043; doi:10.1099/mgen.0.001814)
Supplement: Supplementary file 2 [file mgen-12-01814-s003.pdf]

Supplementary Results

In general, if an HMM hit occurred within a GTDB representative genome, there were 1-2 hit occurrences for the same HMM: ~90% of HMM hit instances within a genome were singular, 8.4% had two hits, and only 1.6% of hit instances yielded 3 or more hits within a single genome. This is consistent with the prevalence of bacterial gene duplicates [1]. However, there were several GTDB representatives with a higher degree of intra-HMM hits. For instance, *Dehalococcoidia bacterium* species UBA1127 sp002311595 (accession GCA_913049785.1) had 54 hits for the same phthalate-degrading enzyme HMM (ID Q9ZFQ5). This species was identified from metagenomic analysis of water samples and has a 93.05% complete genome. Additionally, the same HMM had ≥10 hits within 64 other GTDB representatives. Therefore, it is most likely that these include false positive hits.

Supplementary References

1. Gevers D, Vandepoele K, Simillion C, Van de Peer Y. Gene duplication and biased functional retention of paralogs in bacterial genomes. *Trends in Microbiology*. 2004;12(4):148-54. 10.1016/j.tim.2004.02.007.
